# Comparison of Long vs. Short Concave Neck in One‐Piece Implants Inserted 1 mm Subcrestally: A Pre‐Clinical In Vivo Study

**DOI:** 10.1111/clr.70139

**Published:** 2026-05-13

**Authors:** Hadi Antoun, Frank Bonnet, María Permuy Mendaña, Daniele Botticelli, Giovanna Iezzi, Morena Petrini

**Affiliations:** ^1^ Institut de Formation en Chirurgie Implantaire Avancée Paris France; ^2^ Institut de Formation FIDE Cannes France; ^3^ Department of Veterinary Clinical Sciences, Facultade de Veterinaria, Terra Campus Universidade de Santiago de Compostela Lugo Spain; ^4^ Ibonelab Lugo Spain; ^5^ ARDEC Academy Rimini Italy; ^6^ Department of Medical, Oral and Biotechnological Sciences University G. d'Annunzio of Chieti Chieti Italy

**Keywords:** dental implants, implant neck design, peri‐implant tissues

## Abstract

**Objectives:**

The aim was to assess the influence of one‐piece implant concave neck height on soft‐ and hard‐tissue healing on a canine model.

**Material and Methods:**

Monoblock implants with a transgingival concave neck were randomly inserted 1 mm subcrestally. Eight implants were characterized by a neck 2.8 mm long (group long) and eight implants by 1.8 mm (group short). After 3 months, the implants were retrieved and processed for histology. The quality and quantity of peri‐implant tissues were investigated.

**Results:**

Histological analysis showed that all implants were osseointegrated, and peri‐implant mucosa surrounded the abutment's surface in all 16 specimens. The qualitative analysis of soft tissues showed some differences among the groups. In particular, in the group “long”, collagen fibers perpendicular to the implant's longitudinal axis were intertwined with the transverse fibers. Perpendicular fibers were detected in the same area as the short‐neck implants, but the longitudinal fibers were less numerous and farther from the surface. No statistically significant differences were found in comparisons of soft and hard tissues between the short and long groups.

**Conclusions:**

After 3 months, no statistically significant quantitative differences were found between long and short concave necks for most of the measurements performed, although qualitative differences in collagen fiber orientation were observed.

## 1 Introduction

Maintaining a healthy peri‐implant soft tissue is crucial for long‐term implant success (Berglundh et al. 2007, 2019; Salvi et al. 2015; Tomasi et al. 2014).

Implant transmucosal contouring can substantially influence the development of supracrestal soft tissue and the response of crestal bone throughout both the initial and subsequent stages of treatment, and this concept applies to both two‐piece and one‐piece implants. The “paradigm shift” proposed by Touati et al. (2005) showed that peri‐implant soft tissues could be dramatically improved through the use of transmucosal components with an altered design. The authors suggested the substitution of conventional abutments with divergent walls with convergent, narrow, and concave negative profiles to induce thicker, more stable, and tighter peri‐implant mucosa (Touati et al. 2005).

However, two‐piece and one‐piece implants are not biologically and mechanically equivalent, because the latter lacks the microgap at the implant/abutment interface, which represents a weak point due to micromovements and potential bacterial infiltration (D'ercole et al. 2022). Also, the healing process is different, indeed. After non‐submerged implant installation, the healing process encompasses the intraosseous and transmucosal regions. Mucosal healing involves the formation of a seal around the implant neck, composed of connective tissues and epithelium (Berglundh et al. 2007; Salvi et al. 2015; Tomasi et al. 2014).

The histological analysis of samples from animal models has been highly correlated with the clinical performance of dental implants in humans (Tomasi et al. 2014). In particular, beagle dogs are a preferred model for histology studies in periodontology due to their similarity to human periodontal disease, the ability to induce and study the disease under controlled conditions, and their suitability for detailed histological and regenerative studies (Kim et al. 2010; Kortegaard et al. 2008). Kim et al. (2010) compared the early response of three different types of one‐piece implants on peri‐implant tissues after 6 months of non‐functional loading in Beagle dogs. Results showed that implants with a concave transmucosal profile and a microgrooved surface exhibited the greatest connective tissue attachment, the least marginal bone resorption, and bone growth above an initial reference point, compared with other implants with flared or straight transmucosal portions (Kim et al. 2010).

Bolle et al. (2015) studied early peri‐implant tissue healing on one‐piece implants with a concave transmucosal design inserted 1 mm subcrestally (Bolle et al. 2015). After 12 weeks, a soft tissue O‐ring seal was observed around the transmucosal concavity, the mean soft tissue mucosa was 2.52 mm, and the first bone‐to‐implant contact was +0.18 mm. The authors concluded that the concave transmucosal design promoted mechanical interlocking of the connective tissue at the implant neck and preserved marginal bone levels (Bolle et al. 2015).

A retrospective study in humans showed that in one‐piece implants with a smooth concave neck, the formation of a mucosal attachment to the implant does not appear to occur at the expense of the bony tissue (Axiotis et al. 2018). In particular, the authors found only 0.57 mm of peri‐implant bone loss after 5 years. A recent review confirmed that implants exhibit lower marginal bone loss when characterized by a concave/convergent transmucosal profile than those with a divergent or parallel profile (Valente et al. 2020). All these studies confirmed the positive effects of a concave implant neck on peri‐implant tissue; however, the influence of the height of the concave neck on marginal implant healing has yet to be studied. Therefore, the primary outcome of this study was to assess the histological response of soft‐ and hard peri‐implant tissues to two implant concave necks with different heights.

## 2 Materials and Methods

### 2.1 Ethical Statements

The experimental procedures adhered to the guidelines set out in Directive 2010/63/EU on the ethical treatment of animals used for scientific purposes. The study protocol was approved by the Ethical Committee of the Rof Codina Foundation and the relevant regional authority (Xunta de Galicia, Xefatura Territorial da Consellería do Medio Rural. Ronda da Muralla, 70. Lugo, Spain), under approval number 01/20/LU‐001. Furthermore, the experimental study complied with the ARRIVE guidelines and with institutional and national guidelines for the care and use of laboratory animals, Directive 2010/63/EU (Percie du Sert et al. 2020).

### 2.2 Sample Size and Experimental Animals

Given that the prosthetic platform of the short‐neck implants would be positioned 1 mm closer to the bone crest than that of the long‐neck implants, we anticipated a higher rate of bone crest resorption of 1 mm in the short‐neck group. We assumed that the standard deviation would fall within this range of difference in bone loss; a standard deviation of 0.70 mm was chosen as a reasonable estimate. A sample size of 7 matched pairs was determined to achieve a statistical power of 0.90 and a type I error probability of 0.05. The study ultimately included eight animals to account for potential issues, such as implant loss, and provided a safety margin.

### 2.3 Study Design

Bilateral extraction of the mandibular premolars, as well as the first and second molars, was performed on eight Beagle dogs. The dogs recruited for the present study were admitted to the facility for the experiment by the supplier (Isoquimen SL, Spain) and assigned a protocol code upon arrival. Animals supplied met the inclusion criteria: (a) older than 12 months, (b) all definitive dental pieces erupted, and (c) no missing teeth.

After a 3‐month healing period, one‐piece implants from each group were randomly placed in the molar and premolar regions. Monoblock implants, 3.6 × 8.0 mm with a transgingival, concave, anodized neck (Biotech Dental, France), were used in this study.

Implant allocation followed a randomized split‐mouth design: on one side, a short‐neck implant was placed, and on the other, a long‐neck implant. Consequently, all included dogs received both types of implants, one on each side. The coronal margin of the rough endosseous portion of the implant (R) was positioned 1 mm subcrestally relative to the buccal crest. A periodontal probe was used to check the correct placement of the implant 1 mm subcrestally, starting from point R.

### 2.4 Randomization and Allocation Concealment

An independent individual (D.B.), who was not involved in the surgical procedures, conducted the randomization electronically using Microsoft Excel (Office 365, version 2502). Treatment allocations were placed in sealed, opaque envelopes coded to ensure blinding. The author opened these envelopes immediately after elevating the first flap at implant installation.

One author (G.I.) performed histological measurements and was blinded to the study protocol. Coded histological slides were used to minimize observer bias. However, the difference in neck height was still easily recognizable.

### 2.5 Implant Characteristics

**FIGURE 1:**
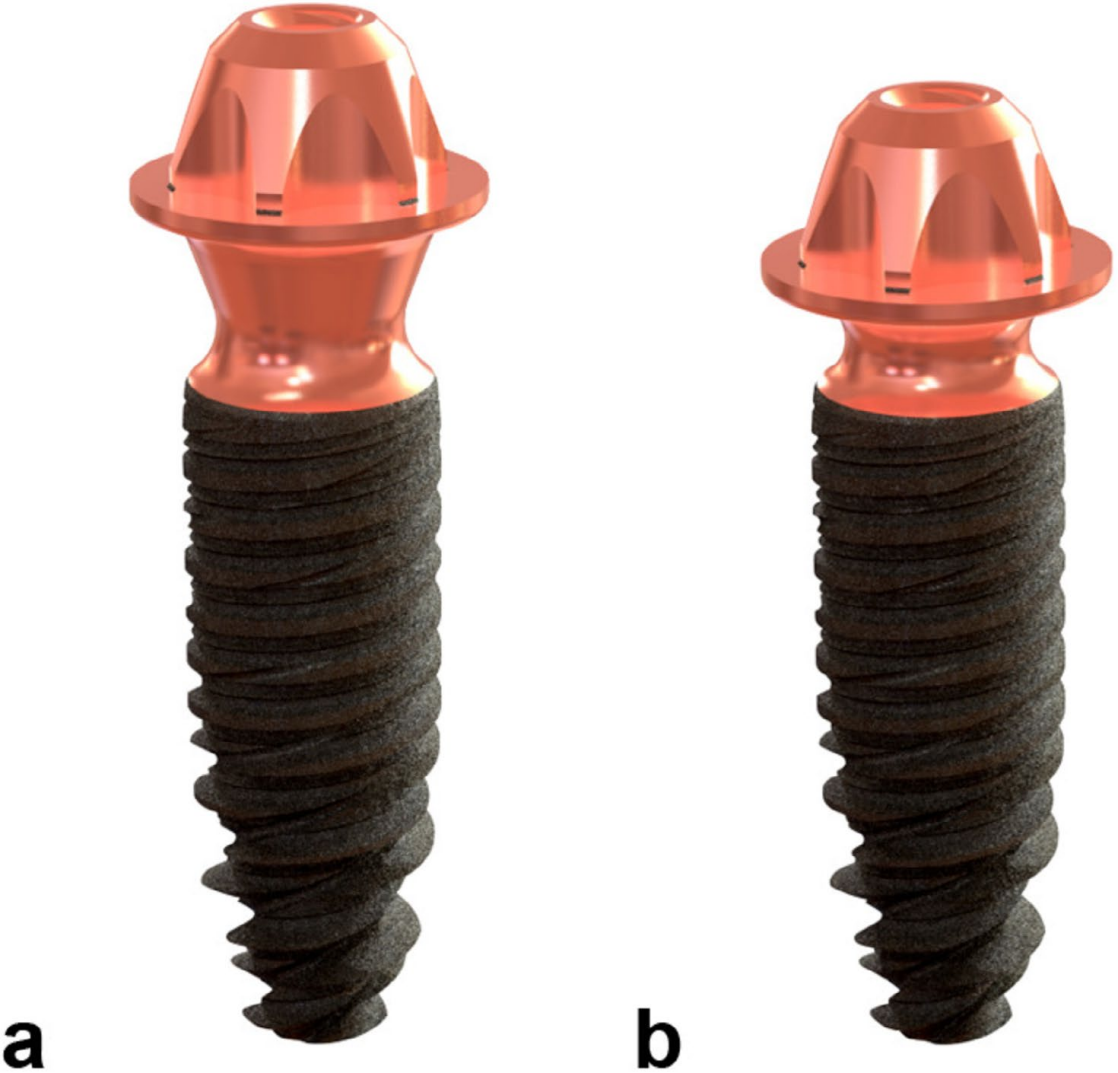
Schematic representation of the two monophasic implants compared in this study: Long neck (a) and short neck (b).

The implant surfaces were made of Grade IV Titanium (T60) and were externally sandblasted and etched. The connection featured a conical abutment MUA (Multi‐Unit Abutment), as shown in Figure 1.

All sixteen implants used in this study were 3.6 × 8.0 mm and were distinguished into two groups of equal sample size (*n* = 8), based on the neck length:

− “group long”: characterized by a 2.8 mm implant's neck (Figure 1a).
− “group short”: characterized by a 1.8 mm implant's neck (Figure 1b).

To simplify the text, implants in the short‐neck group will be referred to as “short” from now on, while those in the long‐neck group will be referred to as “long.”

### 2.6 Anesthesia and Medication Procedures

To prepare the animals, premedication was administered with a combination of medetomidine (10 μg/kg, i.m., Sededorm 1 mg/mL, Vetpharma Animal Health, Barcelona, Spain) and morphine (0.3 mg/kg, i.m., Morfina Braun 2%, B. Braun Medical, Barcelona, Spain). Anesthesia was induced using 2 mg/kg of intravenous propofol (Propofol Lipuro 10 mg/mL, B Braun, Melsungen, Germany), and maintained with 1%–1.5% isoflurane in oxygen (Vetflurane 1000 mg/g, Virbac SA, Carros, France). Vital signs were continuously monitored, including heart rate, respiratory rate, blood pressure, and CO_2_ levels. Initial readings were taken every 5 min after induction, then every 15 min thereafter.

### 2.7 Surgical Procedure

**FIGURE 2:**
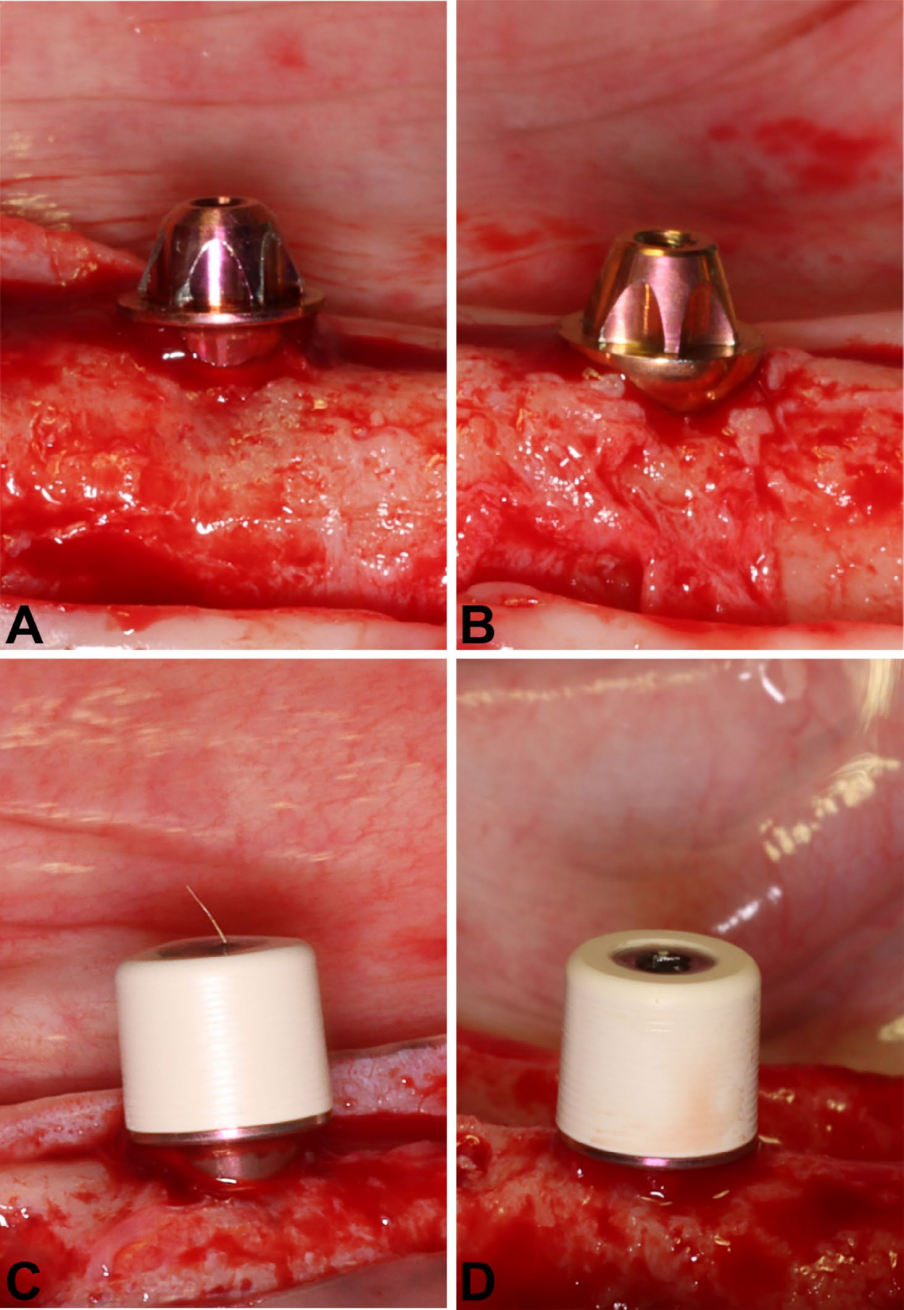
Representative photograph of the surgical phase: Implants immediately after the insertion (long neck a, short neck b) and implants after the insertion of the healing caps and the suturing phase (long neck c, short neck d).

After anesthesia, small flaps were raised, and all premolars and first and second molars were extracted bilaterally. After 3 months, a flap was elevated, and a single implant, either with a long neck (Figure 2a) or a short neck (Figure 2b), was randomly installed on each side of the molar/premolar region of the mandible. Healing caps (Figure 2c,d) were attached to the implants (Kontact Healing cap, 4 mm or 6 mm for MUA conical abutment, Biotech, France), and interrupted sutures (Vicryl 4–0) were used to secure the surgical site and facilitate non‐submerged healing.

### 2.8 Housing and Husbandry

For plaque control, the teeth were cleaned using gauze soaked in 0.12% chlorhexidine three times a week during the first 2 weeks. Afterward, the protocol was switched to brushing the teeth three times weekly with a toothbrush and chlorhexidine gel, and this practice continued until the end of the study.

Postoperative pain management was provided with buprenorphine (0.01 mg/kg, i.m., every 8 h, Bupaq, Richter Pharma AG, Wells, Austria). Meloxicam (Loxicom, Norbrook Laboratories, Monaghan, Ireland) was given intravenously before surgery (0.2 mg/kg) and then orally for 2 days (0.1 mg/kg, p.o., once daily). Prophylactic antibiotics, including cefazolin (20 mg/kg, i.v., Cefazolina Normon, Madrid, Spain) and sodium cefovecin (8 mg/kg, s.c., once daily, Convenia, Zoetis, Madrid, Spain), were administered before each surgical procedure.

No adverse effects were detected, and therefore, no dropouts were reported.

### 2.9 Euthanasia

Three months after the final surgery, the dogs were sedated and then humanely euthanized using a lethal dose of sodium pentobarbital (200 mg/kg, i.v., Dolethal, Vetoquinol, France).

All the implants were retrieved using a 5‐mm trephine bur and then immersed in 10% buffered formalin for histological processing.

### 2.10 Histological Preparation

The samples were managed to obtain thin ground sections with the Precise 1 Automated System (Assing, Rome, Italy). The specimens were dehydrated in an ascending series of alcohol rinses and embedded in a glycol methacrylate resin (Technovit 7200 VLC; Kulzer, Wehrheim, Germany). After polymerization, the specimens were sectioned into two portions along their longitudinal axis in the bucco‐lingual direction using a high‐precision diamond disk. The inner mesial portion of the sample was sectioned at approximately 150 μm and then ground to approximately 30 μm with a specially designed grinding machine (Canullo et al. 2023).

**FIGURE 3:**
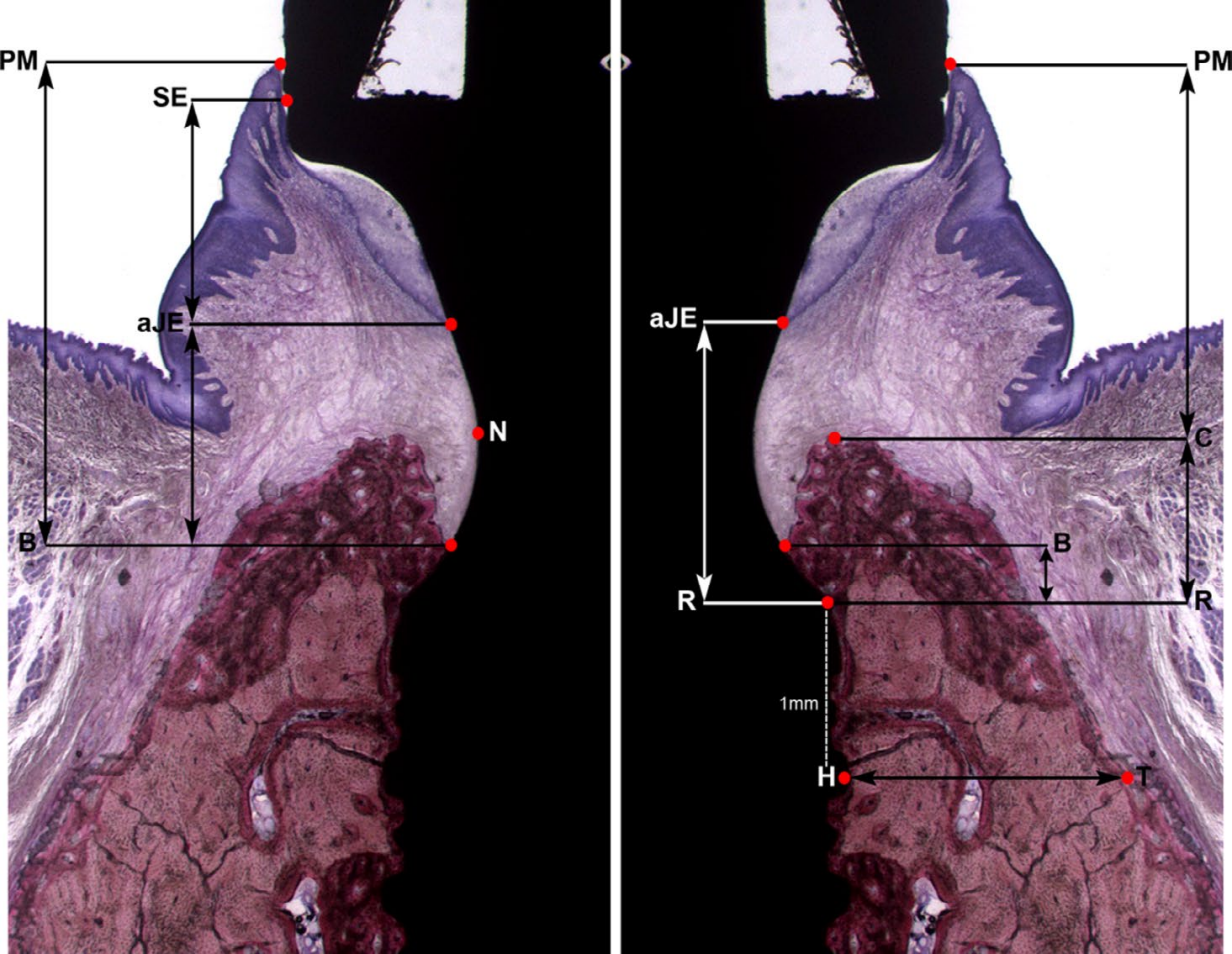
Schematic slide describing the points from which histometric measurements were taken. On the left, the points and the distance are used to identify the soft tissues. On the right, the points are used to identify the different landmarks of the hard tissues.

The distal portion of the samples was used to obtain a transversal section perpendicular to the implants' long axis. The section was performed at the narrowest portion of the neck, at point N for each implant (Figure 3).

Histological analysis was conducted using a light microscope (Laborlux S; Leitz) equipped with a high‐resolution video camera (3CCD, JVCKY‐F55B; JVC) and interfaced with a personal computer. This optical system was associated with a digitizing pad (Matrix Vision GmbH) and a histomorphometry software package with image‐capturing capabilities (Image‐Pro Plus 4.5; Media Cybernetics Inc., Immagini and Computer Snc). Histological images were obtained at 40× and 100× magnification to facilitate histological evaluations. One single well‐trained examiner (GI), not involved in the surgical treatment, evaluated the histological results. All histological and histomorphometric measurements were performed according to a predefined, standardized protocol and under blinded conditions, using coded histological sections. The examiner's calibration was verified by calculating the intraclass correlation coefficient (ICC) in SPSS Statistics for Windows, version 21 (IBM SPSS Inc., Chicago, IL, USA), which yielded a value greater than 0.9.

Qualitative observations obtained by polarized light microscopy were descriptive.

### 2.11 Histomorphometric Evaluation

All histological sections were evaluated by a trained expert examiner (G.I.) who identified the following points (Figure 3):

- the first bone‐implant contact (B)
- the top of the bone crest (C)
- the original bone crest position, 1 mm coronally to R (OC)
- the top of the peri‐implant mucosa (PM)
- the apical portion of the sulcular epithelium (SE)
- the apical point of the junctional epithelium (aJE)
- the narrowest portion of the neck (N)
- the coronal margin of the rough surface (R)
- the point H situated 1 mm under the point R
- the point T that is the projection of point H on the buccal and lingual cortical

Then, the following measurements were carried out:

- the distance between PM and C, corresponding to the height of the soft tissues (PM‐C)
- the distance between point PM and B corresponds to the distance between the top of the peri‐implant mucosa to the first bone‐to‐implant contact (PM‐B)
- the distance between PM and R (PM‐R)
- the distance between PM and the original bone crest position OC (PM‐OC)
- the distance between aJE and B, corresponding to the height of the connective tissue (aJE‐B)
- the distance between aJE and R, corresponding to the height of the connective tissue comprised between the coronal portion of the rough surface and the junctional epithelium (aJE‐R)
- the distance between SE and aJE corresponding to the linear height of the junctional epithelium (JE)
- the position of the first BIC from original crest (OC‐B), corresponding to the marginal bone loss
- the position of bone crest from original crest (OC‐C), corresponding to the crestal bone loss
- the distance between the R and C (R‐C)
- the distance between point C and B (C‐B), corresponding to the marginal defect
- the distance between point R and first BIC B (R‐B); results were expressed as positive if point B was more coronal with respect to point R and negative in the case of point B being more apical
- the distance between T and H, corresponding to the thickness of the bone crest measured 1 mm under the point R (T‐H)

As previously described, these distances were measured as vertical distances in a direction parallel to the implant's long axis (Tomasi et al. 2014). Results were expressed as positive if the first point of each segment was more coronal than the second.

In addition to the linear measurements, profile measurements were performed for junctional epithelium (JEp) and the height of the connective tissues (aJE‐Bp). These “true” distances of “profile” dimensions were assessed by outlining the soft tissue interface toward the implant neck using a mouse cursor (Tomasi et al. 2014). The measurements were recorded in μm with 3 decimal numbers.

### 2.12 Polarized Light

Polarized light microscopy was used to visualize collagen orientation via birefringence, both on longitudinal section and transversal section, for each sample. Collagen fibers were viewed by placing thin tissue sections under an Axiolab light microscope (Laborlux S; Leitz) equipped with two linear polarizers and two quarter‐wave plates to transmit circularly polarized light. Collagen fibers aligned perfectly transverse to the direction of the light propagation (parallel to the plane of the section). They appeared bright due to a change in the refraction of existing light. In contrast, the collagen fibers aligned along the axis of light propagation (perpendicular to the plane of the section) appeared with different colors because no refraction occurred.

### 2.13 Statistical Analysis

The software SPSS Statistics for Windows, version 21 (IBM SPSS Inc., Chicago, IL, USA), was used to compare intragroup and intergroup analyses of quantitative variables. A *p*‐value < 0.05 was considered significant.

The Levene Test verified the homogeneity of the samples before the *T*‐test. The groups were considered homogeneous if Levene's test statistic was not significant (*p* > 0.05).

The statistical analysis was also performed at the implant level by averaging buccal and lingual measurements per implant, and an intergroup analysis was then performed comparing the “short” vs. “long” groups (Table 1).

**TABLE 1:** Histometric data of hard and soft tissues around the implant necks, at the implant level.

Parameter	Group	Mean values (mm)	SD	Levene Sig.	*T*‐test
PM‐C	Short	1.95	0.28	0.09	0.82
	Long	2.00	0.50		
PM‐B	Short	2.47	0.60	0.92	0.21
	Long	2.90	0.69		
PM‐R	Short	2.46	0.58	0.65	0.16
	Long	2.95	0.76		
PM‐OC	Short	1.43	0.60	0.70	0.17
	Long	0.94	0.76		
aJE‐B	Short	0.96	0.41	0.32	0.09
	Long	1.27	0.25		
aJE‐R	Short	0.95	0.35	0.93	0.05
	Long	1.32	0.32		
JE	Short	1.10	0.55	0.69	0.73
	Long	1.19	0.51		
OC‐B	Short	1.04	0.48	0.25	0.68
	Long	0.97	0.19		
OC‐C	Short	0.54	0.53	0.69	0.09
	Long	0.06	0.52		
C‐B	Short	0.53	0.40	0.83	0.09
	Long	0.91	0.43		
R‐B	Short	0.16	0.47	0.28	0.72
	Long	−0.05	0.20		
R‐C	Short	−0.51	0.49	0.90	0.10
	Long	−0.96	0.52		
T‐H	Short	1.83	0.50	0.04	0.81
	Long	1.78	0.23		
aJE‐Bp	Short	1.26	0.49	0.16	0.37
	Long	1.45	0.31		
JEp	Short	1.90	0.53	0.67	0.37
	Long	1.62	0.71		

*Note:* Mean values ± standard deviations were expressed in mm. Intergroup analysis: Levene Test significance and *T*‐test. *p*‐value < 0.05.

Then, the following comparisons were also performed:

- Intergroup analysis of buccal side measurements on group “short” vs. “long” (Table 2)
- Intergroup analysis of lingual side measurements on group “short” vs. “long” (Table 2)

**TABLE 2:** Histometric data of hard and soft tissues around the implant necks.

Parameter	Group	Buccal				Lingual			
		Mean values (mm)	SD	Levene Sig.	*T*‐test (2‐code)	Mean values (mm)	SD	Levene Sig.	*T*‐test (2‐code)
PM‐C	Short	2.54	0.47	0.19	0.59	1.35	0.35	0.54	0.73
	Long	2.71	0.73			1.28	0.50		
PM‐B	Short	3.01	0.95	0.87	0.58	1.94	0.79	0.51	0.20
	Long	3.25	0.74			2.55	1.03		
PM‐R	Short	2.76	0.57	0.71	0.13	2.16	0.87	0.70	0.35
	Long	3.27	0.70			2.63	1.07		
PM‐OC	Short	1.74	0.59	0.78	0.14	1.13	0.89	0.76	0.34
	Long	2.26	0.71			1.62	1.07		
aJE‐B	Short	1.22	0.66	0.22	0.49	0.70	0.24	0.38	0.03*
	Long	1.41	0.30			1.14	0.41		
aJE‐R	Short	0.98	0.29	0.30	0.01*	0.93	0.43	0.67	0.20
	Long	1.43	0.35			1.22	0.42		
JE	Short	1.23	0.53	0.70	0.94	0.97	0.78	0.76	0.67
	Long	1.25	0.60			1.14	0.71		
OC‐B	Short	1.28	0.68	0.42	0.33	0.81	0.49	0.26	0.53
	Long	1.00	0.33			0.93	0.22		
OC‐C	Short	0.80	0.35	0.22	0.10	0.28	0.77	0.51	0.12
	Long	0.46	0.42			−0.34	0.74		
C‐B	Short	0.47	0.58	0.13	0.77	0.58	0.58	0.91	0.05*
	Long	0.54	0.23			1.28	0.70		
R‐B	Short	0.26	0.68	0.47	0.33	−0.23	0.48	0.31	0.46
	Long	−0.02	0.34			−0.08	0.22		
R‐C	Short	−0.21	0.33	0.15	0.09	−0.81	0.69	0.73	0.14
	Long	−0.56	0.42			−1.36	0.74		
T‐H	Short	1.41	0.56	0.54	0.33	2.25	0.53	0.35	0.54
	Long	1.16	0.41			2.40	0.41		
aJE‐Bp	Short	1.60	0.85	0.08	0.99	0.92	0.26	0.26	0.07
	Long	1.59	0.38			1.30	0.49		
JEp	Short	2.23	0.62	0.43	0.26	1.58	0.92	0.86	0.74
	Long	1.82	0.78			1.42	1.02		

*Note:* Mean values ± standard deviations were expressed in mm. Intergroup analysis: Levene Test significance and *T*‐test.

* *p*‐value < 0.05.

The statistical analysis was carried out on measurements expressed in μm with three decimal places; however, results in the text and Tables are reported in mm and rounded to two decimal places.

## 3 Results

### 3.1 Histological Analysis

#### 3.1.1 Implants With Short and Long Necks

**FIGURE 4:**
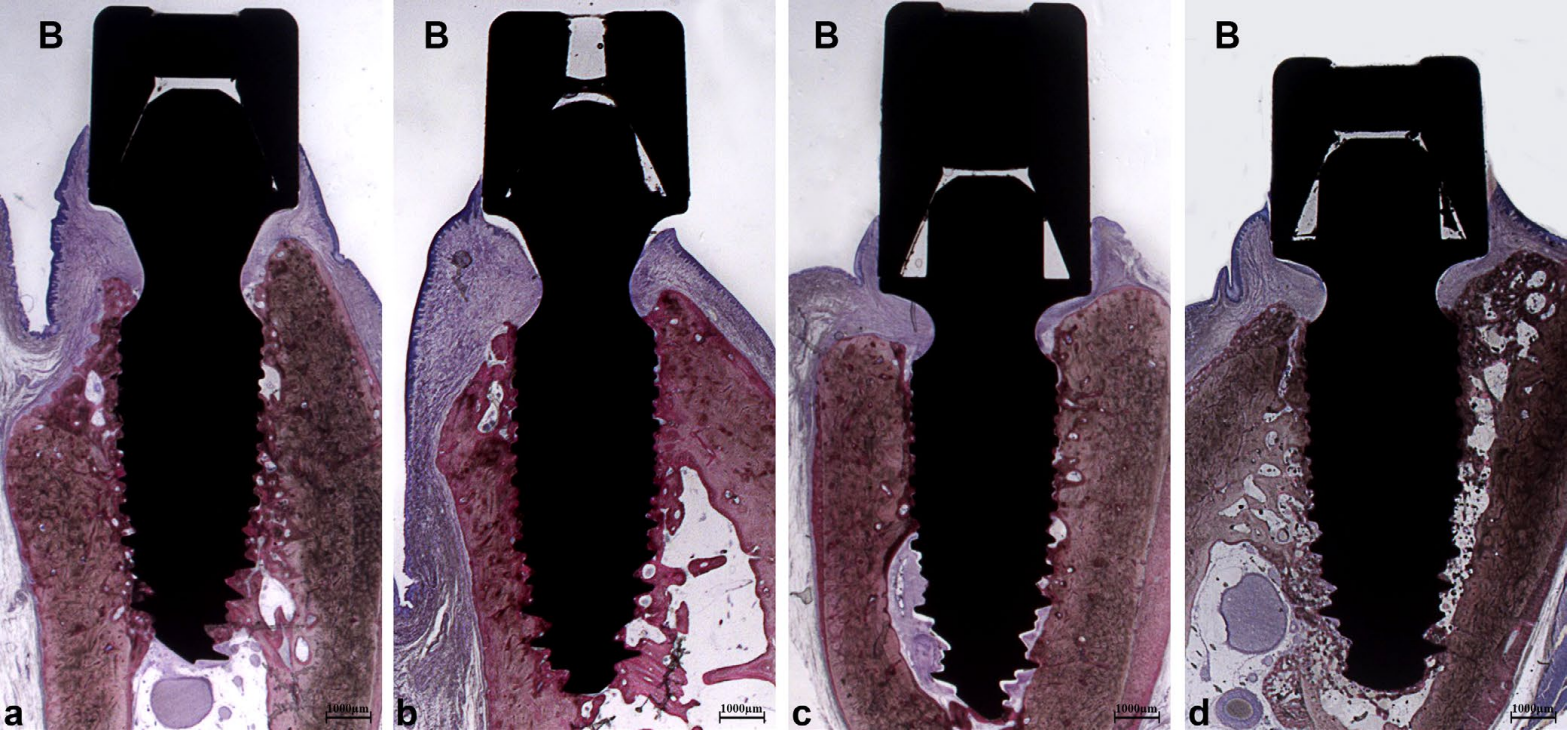
Bucco‐lingual section after 3 months of healing, showing implants with a long neck (a, b) and Implants with a short neck (c, d). The buccal side is indicated by the letter B. In all implants, the buccal bone wall is thinner and more apical than the lingual bone wall. The peri‐implant mucosa surrounded the abutment surface in all specimens. Regarding peri‐implant bone healing, all implants were osseointegrated (a–d) (Acid fuchsin‐Toluidine blue 3×).

Histological analysis showed that peri‐implant mucosa surrounded the abutment surface in all 16 specimens. Furthermore, all implants were osseointegrated, as they demonstrated close bone‐to‐implant contact (Figure 4).

**FIGURE 5:**
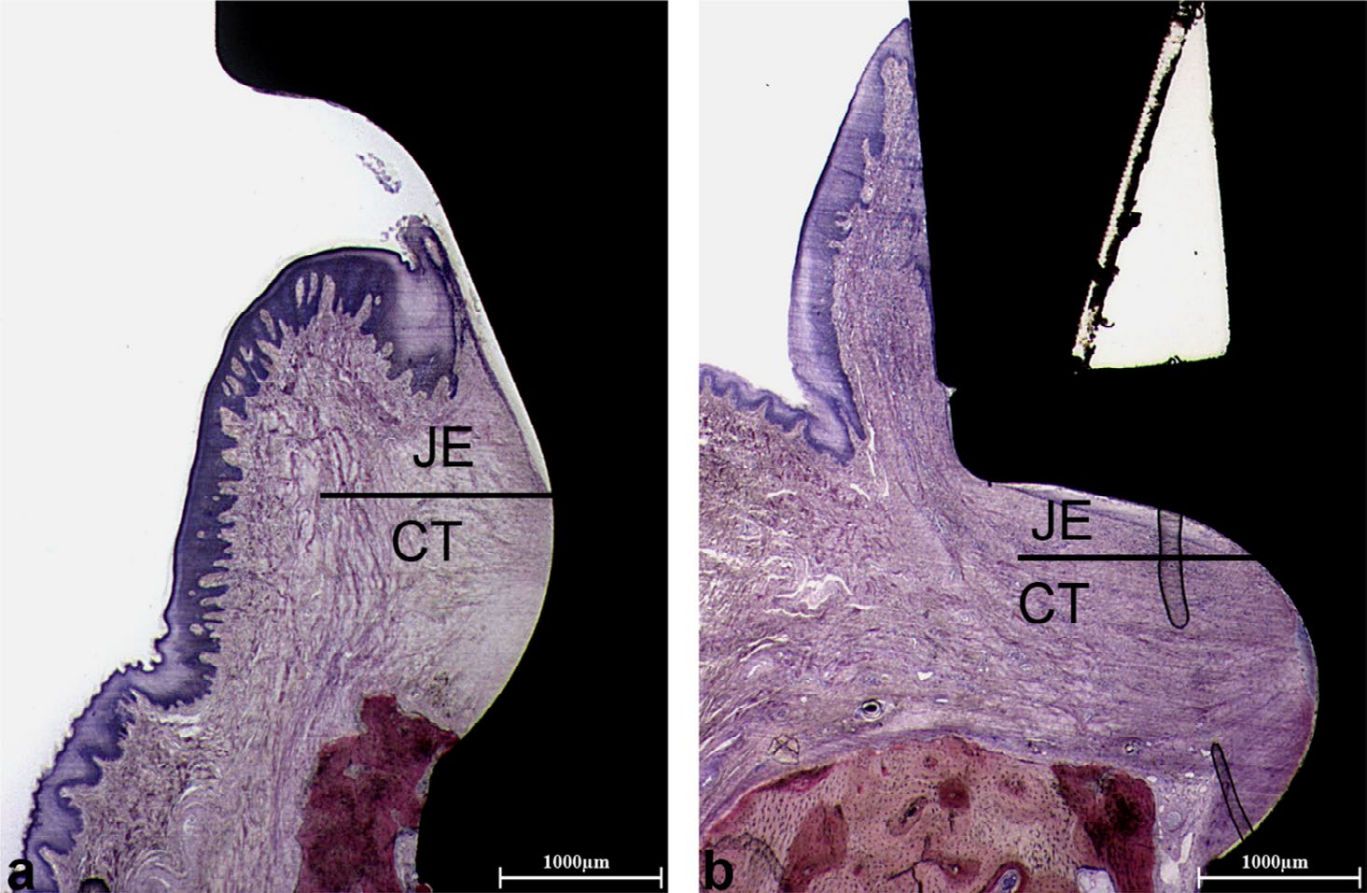
Buccal area of interest showing the soft tissues around implants with long (a) and short neck (b). Implants with short necks exhibited a small gap between the junctional epithelium (JE) and the abutment surface, likely due to the removal procedure. In both groups, a gap was never observed between the connective tissue (CT) and the surface of the abutments (Acid fuchsin‐toluidine blue 12×).

Peri‐implant mucosa analysis revealed that in some specimens, a small gap was found between the junctional epithelium and the abutment surface, likely caused by the removal procedure. As shown in Figure 5, the different heights of the implant necks that distinguish the two groups translate into different concavities.

The neck of the short group showed greater concavity than that of the long group. Tissue maturation and collagen fiber organization were evident after 3 months of healing; furthermore, the formation of junctional epithelium and connective tissue was well represented in all specimens (Figure 5). Indeed, the connective tissue showed signs of organization, with numerous fibroblasts arranged along the axis of the collagen fibers. Regardless of the groups, a modest inflammatory infiltrate was observed in several specimens, mainly localized at the junctional epithelium.

Analyzing the behavior of the crestal bone, histology revealed bone remodeling and partial crestal bone loss around all implants, more evident around those with short necks (Table 1).

**FIGURE 6:**
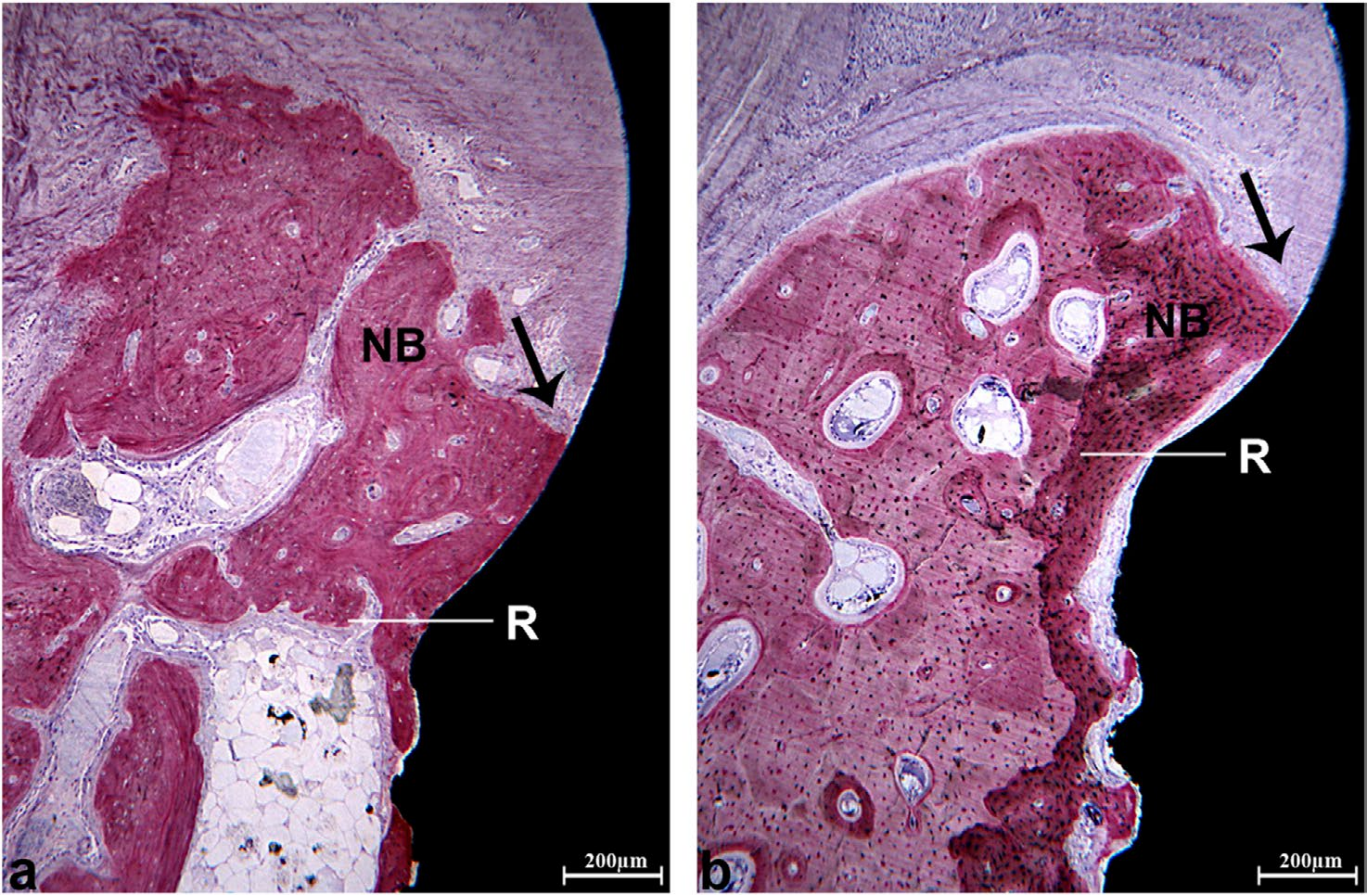
Buccal area of interest showing the bone crest around implants with long (a) and short necks (b). The buccal bone plate is composed predominantly of newly formed bone (NB) in its most coronal aspect. Buccal bone remodeling was present in two types of implant systems. The bone was present above the rough portion (R) of the implant and in close contact with the neck surface (black arrow) (a, b) (Acid fuchsin‐toluidine blue 40×).

However, in some samples, the bone was present above the rough portion of the implant and in close contact with the neck surface. In no case was the crestal bone colonized by multinucleated giant cells. On the contrary, new bone formation was observed after a 3‐month healing period, even around the implants where initial bone resorption may have occurred (Figure 6).

#### 3.1.2 Polarized Light of Peri‐Implant Mucosa

**FIGURE 7:**
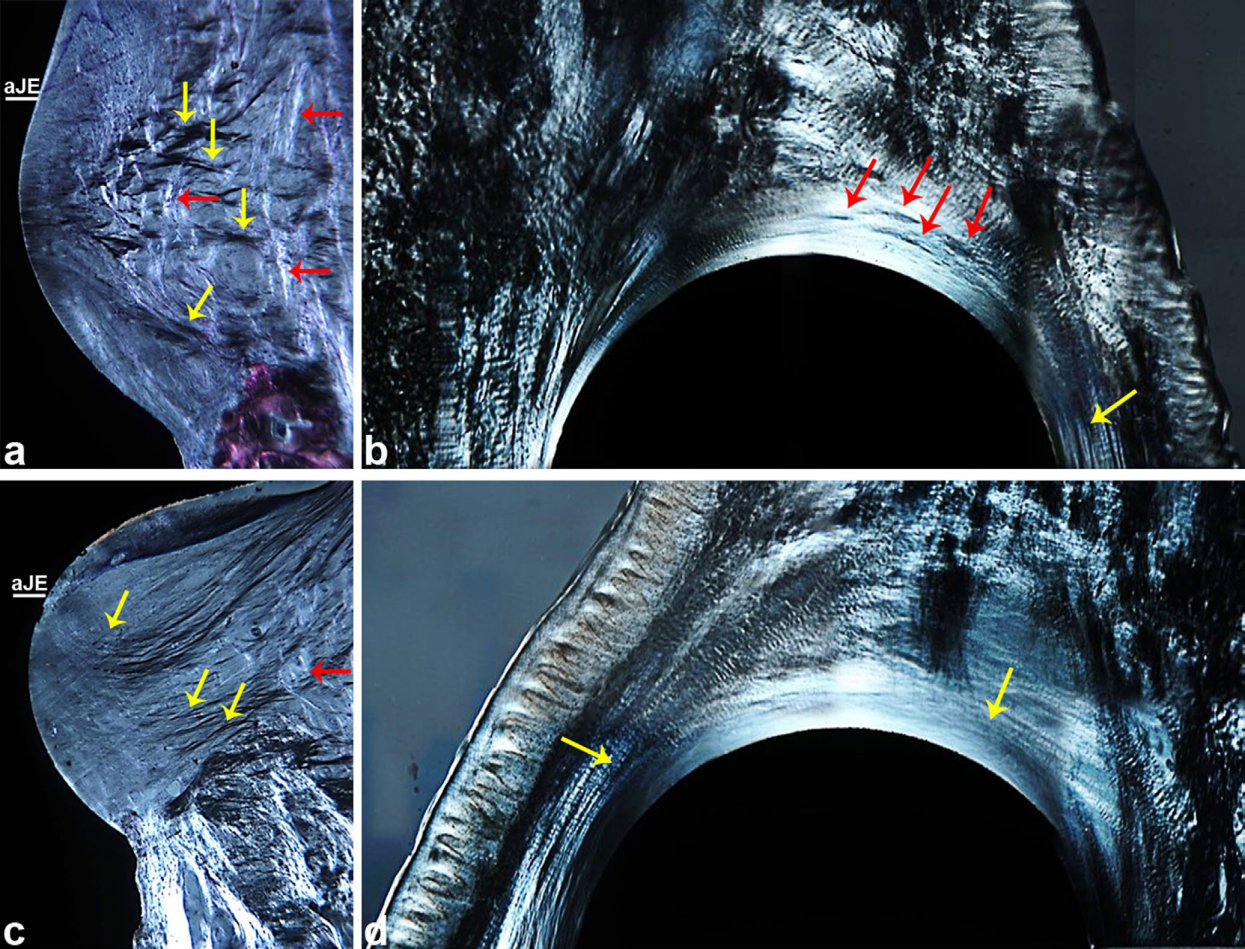
Polarized light microscopy of the peri‐implant soft tissues. Longitudinal section of the buccal peri‐implant soft tissue of implants with a long neck (a). The transversal collagen fibers ran perpendicular to the longitudinal axis of the implant toward the concavity of the neck. Some transverse fibers appear to converge with each other, forming thicker bundles (yellow arrows), while the longitudinal fibers (red arrows) intertwine with the transverse fibers. The portion where the transverse collagen fibers converge is generally the point where the apical extension of the junctional epithelium (JE) is observed (a). Transversal section of the peri‐implant soft tissue of implants with a long neck (b). Collagen fibers ran close to the circular profile of the concavity (yellow arrows). However, in small areas, the fibers appeared differently colored because no refraction occurred (red arrows). These areas probably correspond to the longitudinal fibers observed in the longitudinal section (b). Longitudinal section of the buccal peri‐implant soft tissue of implants with a short neck (c). Numerous transverse collagen fibers were observed running perpendicular to the longitudinal axis of the implant toward the entire concavity of the neck (yellow arrows). Longitudinal fibers were also present, however they were less numerous and further from the surface (red arrow) (c). Transversal section of the peri‐implant soft tissue of implants with a short neck (d). Collagen fibers run close to the circular profile of the concavity. These showed a homogeneous semicircular orientation (yellow arrows) (d).

Polarized light microscopy revealed that the peri‐implant mucosa consisted of several collagen bundles, distinguishable in both longitudinal and transverse sections, and differences in the distribution of collagen fibers were observed between the groups (Figure 7).

### 3.2 Long Neck

#### 3.2.1 Longitudinal Section

Peri‐implant mucosa analysis revealed transverse collagen fibers running perpendicular to the implant's longitudinal axis toward the neck's concavity. In the same area, longitudinal fibers were found to be intertwined with the transverse fibers.

The portion where the transverse collagen fibers converged was generally the point where the apical extension of the junctional epithelium was observed (Figure 7a).

#### 3.2.2 Transversal Section

In the transversal section obtained in the narrowest portion of the neck, the collagen fibers ran close to the circular profile of the concavity. However, in small areas, the collagen fibers appeared with different colors because no refraction occurred. These areas likely correspond to the longitudinal fibers highlighted in the Longitudinal section (Figure 7b).

### 3.3 Short Neck

#### 3.3.1 Longitudinal Section

When analyzing the peri‐implant mucosa, numerous transverse collagen fibers were observed running perpendicular to the longitudinal axis of the implant, extending toward the entire concavity of the neck. The longitudinal fibers were fewer in number and farther from the surface.

The portion where the transverse collagen fibers converged was generally the point where the apical extension of the junctional epithelium was observed (Figure 7c).

#### 3.3.2 Transverse Section

In the transverse section obtained at the narrowest portion of the neck, the collagen fibers running close to the circular profile of the concavity exhibited a homogeneous, semicircular orientation. In contrast, in the more distant portion, they were less identifiable (Figure 7d).

### 3.4 Quantitative Analysis

The statistical analysis at the implant level (Table 1) showed no significant differences between the short and long groups. Group long showed higher values for soft tissue measurements, such as PM‐B, PM‐R, AjE‐B, and AjE‐R, but these differences were not statistically significant compared with group short. Crestal bone loss OC‐C was higher in the short group, but without statistically significant differences between the two groups. The marginal defect was higher in the long group.

On the buccal side, all soft‐tissue measurements were higher in the long group; however, aJE‐R was the only parameter with a statistically significant difference (*p* = 0.01; Table 2).

No statistically significant differences in hard tissues were found between the short and long groups.

However, group short showed higher values of marginal bone loss (OC‐B) and crestal bone loss than group long (OC‐C).

In the lingual side (Table 2), statistically significant differences were found for AjE‐B and C‐B. The height of the connective tissues (aJE‐B) and the marginal defect were significantly higher in group long (*p* < 0.05).

## 4 Discussion

Information on long‐term clinical outcomes and implant longevity can be obtained by evaluating soft‐ and hard‐tissue quality and quantity. Soft tissue concerns are critical to the long‐term success of dental implants, influencing both function and appearance (Sharma et al. 2024). The main goal of this study was to compare peri‐implant tissues of two monoblock implants that differ in neck length and were inserted 1 mm subcrestally. Both types of implants had a concave neck, but group “short” was characterized by a higher concavity than group “long”. The histological analysis of soft and hard peri‐implant tissues has been performed, and the data have been analyzed.

The three‐dimensional network of collagen fibers surrounding the implant neck is important because it may provide mechanical protection to the underlying bone, stabilize soft tissues, and prevent their apical migration (Degidi et al. 2012). In this study, qualitative analysis of soft tissues performed using polarized light microscopy showed that the group long was characterized by a more intertwined conformation, with longitudinal and transverse collagen fibers detected at similar percentages. On the contrary, in the short group, transverse fibers were more prevalent; longitudinal fibers were less numerous. The statistical analysis performed at the implant level (Table 1) showed no significant differences between the short and long groups. However, the group long showed higher values for soft‐tissue measurements, such as PM‐B, PM‐R, AjE‐B, and AjE‐R, but these differences were not statistically significant. Crestal bone loss OC‐C was greater in the short group.

A detailed evaluation of buccal and lingual measurements of soft and hard tissues is fundamental for both function and aesthetics (López‐Jarana et al. 2018; Thoma et al. 2014; van Brakel et al. 2011).

Quantitative measurements of the buccal sides of the “short” and “long” groups showed that the latter had higher soft and hard tissue heights. On the contrary, the “short” group was characterized by greater crestal bone thickness. However, although these data are interesting, they were not statistically significant.

Puisys et al. (2024) showed that implants placed in areas with reduced mucosal thickness exhibited greater marginal bone loss after 10 years (Puisys et al. 2024). In a retrospective study, Le and Borzabadi‐Farahani (2012) found a strong correlation between crestal labial soft tissue thickness (CLSTT) and implant labial bone thickness (ILBT). Other authors have highlighted the importance of having at least 2 mm of buccal soft tissue volume to preserve the aesthetics of dental implants (Thoma et al. 2014; van Brakel et al. 2011). No studies on one‐piece implants analyzed the effects of concave neck height on peri‐implant tissues; the only literature was based on two‐piece implants. Spinato et al. showed that implants with short prosthetic abutments placed equicrestally showed more significant marginal bone loss than those with long ones, at 12 months (Spinato et al. 2019). Lombardi et al. (2019) showed that the factors associated with early marginal bone loss were deep implant insertion, thin peri‐implant mucosa, and short abutments after 6 months of prosthetic loading.

Establishing an adequate peri‐implant mucosa is crucial for minimizing bone loss (Zheng et al., 2021). It is typically 3–4 mm, but if it is less than 2 mm, dental implants are more prone to gingival recession and bone loss (Farhoudi and Parsay 2018). In this study, the peri‐implant mucosa (PM‐B) on the buccal side was greater in the “long” group. Indeed, the PM‐B was comprehended between 1.946 and 4.206 mm in the “long” group and 1.991 and 3.546 mm in the “short” group. However, no statistically significant differences have been found between the groups. Also, on the lingual side, the average PM‐B was higher in the “long” group but without statistically significant differences.

The height of the connective tissue (aJE‐B) of the buccal side was 1.41 in group long and 1.22 in group short, in accordance with previous literature that showed that the implant‐mucosal barrier was composed of about 2 mm of epithelium and 1–1.5 mm of connective tissue (Abrahamsson et al. 1996; Berglundh et al. 1991).

The buccal junctional epithelium lengths of both groups were very similar: 1.23 mm in the short group and 1.25 mm in the “long” group. However, the measurement of the “true” JEp showed higher values for the short group but without statistically significant results (Table 2). On the lingual side, a significantly higher value of aJE‐B was found in the long group than in the short group, *p* = 0.03* (Table 2).

After dental extraction, marked hard‐tissue alterations take place both on the buccal and lingual sides of the alveolar bone. However, as shown by Araújo et al. (2005), vertical bone loss is more pronounced at the buccal than at the lingual aspect of the ridge. The buccal bone wall undergoes extensive remodeling, with horizontal resorption ranging from 80% to 63% and vertical resorption from 69% to 65% (Sanz et al. 2010). In this study, the implants were placed in healed sites; however, full‐thickness flap elevation could also have traumatized the delicate buccal bone.

Although no statistically significant differences were observed, the short group exhibited higher values of marginal and crestal bone loss than the long group.

In the lingual aspect, the distance between the bone crest and the first BIC (C‐B) was significantly higher in group long, with respect to group short (*p* < 0.03), Table 2. This value was also confirmed in the implant‐level statistics, but without significant results (*p* = 0.086).

Strong evidence exists between crestal bone height, thickness, and implant stability (Chatvaratthana et al. 2017). In our study, implants with a longer neck exhibited higher mean soft‐ and hard‐tissue heights than those in the short‐neck group. However, the results were not statistically significant. The reason is that although a difference was found between the average values of the measured parameters, significant sample variability was observed, with high standard deviation values. The reason for this considerable variability is that the implants have been inserted in molar and premolar regions of the jaws. The thickness of crestal bone is dependent on the jaw region. Gupta et al. (2017) showed that the posterior region of the mandible is characterized by higher thickness, followed by the anterior mandible, anterior maxilla, and posterior maxilla. However, in this study, the positions of both groups were randomly allocated but also stratified to reduce the influence of anatomical differences.

The limitation of this study is the 3‐month follow‐up; more likely, a longer interval would permit complete hard‐tissue healing, making quantitative analysis measurements statistically significant. Other limitations are a small sample size (8 animals, 16 implants), the absence of radiographic baseline bone measurements, the lack of collagen fiber quantification, and the limited translational value of a preclinical animal model. The lack of statistical significance in quantitative hard‐tissue parameters may reflect biological immaturity rather than a true absence of effect. Indeed, a 3‐month healing period may not adequately capture complete bone remodeling and stabilization of supracrestal tissue attachment. Also, the limited sample size significantly constrains statistical power. Consequently, findings should be interpreted as exploratory rather than definitive, and long‐term clinical extrapolation is premature.

## 5 Conclusion

After 3 months of healing in a canine model, no statistically significant quantitative differences were found between long and short concave necks for most of the measurements performed, although qualitative differences in collagen fiber orientation were observed.

## Author Contributions

**Morena Petrini:** investigation, writing – original draft, writing – review and editing, visualization, validation, methodology, software, formal analysis, data curation. **Frank Bonnet:** writing – review and editing, supervision, validation. **Giovanna Iezzi:** investigation, writing – original draft, writing – review and editing, visualization, validation, methodology, supervision. **María Permuy Mendaña:** writing – review and editing, validation, supervision. **Daniele Botticelli:** conceptualization, investigation, writing – original draft, writing – review and editing, visualization, validation, methodology, software, formal analysis, data curation, funding acquisition. **Hadi Antoun:** conceptualization, investigation, writing – original draft, writing – review and editing, validation, visualization, software, formal analysis, data curation.

## Funding

This study was funded by Biotech Dental, Salon‐de‐Provence, France, who provided all implants free of charge.

## Ethics Statement

The study protocol was approved by the Ethical Committee of the Rof Codina Foundation and the relevant regional authority (Xunta de Galicia, Xefatura Territorial da Consellería do Medio Rural. Ronda da Muralla, 70. Lugo, Spain), under approval number 01/20/LU‐001. Experiments were performed in accordance with relevant institutional and national guidelines for the care and use of laboratory animals (Directive 2010/63/EU), and the experimental study complied with the ARRIVE guidelines.

## Conflicts of Interest

The authors declare no conflicts of interest.

## Data Availability

The data that support the findings of this study are available from the corresponding author upon reasonable request.
